# Vestibular Impairment and Postural Development in Children With Bilateral Profound Hearing Loss

**DOI:** 10.1001/jamanetworkopen.2024.12846

**Published:** 2024-05-23

**Authors:** Sylvette R. Wiener-Vacher, Marta Campi, Simona Caldani, Hung Thai-Van

**Affiliations:** 1 Institut de l’Audition, Institut Pasteur, Centre De Recherche et d'Innovation et Audiologie Humaine (CERIAH), Paris, France; 2 Service ORL, Centre d’Exploration Fonctionnelle de l’Equilibre Chez l’Enfant (EFEE), Hôpital Universitaire Robert Debré, Assistance Publique–Hôpitaux de Paris, Paris, France; 3 Hospices Civils de Lyon, Hôpital Edouard Herriot et Hôpital Femme Mère Enfant, Service d’Audiologie et Explorations Otoneurologiques, Lyon University, Lyon, France

## Abstract

**Importance:**

Children with profound hearing loss (HL) and vestibular impairment have worse cochlear implant outcomes compared with those without vestibular impairment. However, the decision for cochlear implantation is rarely based on vestibular function assessment as a complement to audiologic testing.

**Objectives:**

To identify the prevalence of vestibular impairment according to HL origin and to assess the association between vestibular impairment and delayed posturomotor development in children with profound HL.

**Design, Setting, and Participants:**

This cohort study was conducted in a pediatric referral center for cochlear implantation in Paris, France, using medical records data on HL origin, vestibular assessment, and ages of developmental milestone achievement. The cohort included children with profound HL (loss >90 dB HL) who completed vestibular assessment prior to cochlear implantation between January 1, 2009, and December 31, 2019. Data analyses were conducted between January and June 2023.

**Main Outcomes and Measures:**

The primary outcome was prevalence of vestibular impairment according to HL origin. Children were classified into 3 groups according to their responses to vestibular testing: normal vestibular function (NVF), partially impaired vestibular function (PVF), and complete bilateral vestibular loss (CBVL). Generalized logit models were performed to evaluate the association between vestibular impairment and causes of HL as well as posturomotor development delay.

**Results:**

A total of 592 children were included (308 males [52.0%]; mean [SD] age, 38 [34] months). In children with documented HL origin (n = 266), 45.1% (120) had HL with genetic origin, 50.0% of which were syndromic (mainly Usher and Waardenburg syndromes) and 50.0% were nonsyndromic (mainly associated with connexin 26). Among patients with infectious HL origin (n = 74), 70.3% (52) had cytomegalovirus (CMV) infection. Vestibular impairment was found in 44.4% (263 of 592) of the children; it was mostly symmetrical in 88.9% (526) and was CBVL in 5.7% (34) of the cases. Vestibular impairment was present in 78.3% (47) of children with genetic syndromic HL (56.7% [34] with PVF; 21.7% [13] with CBVL) and in 69.2% (36) of children with CMV infection (57.7% [30] with PVF; 11.5% [6] with CBVL). Genetic syndromic HL origin was found to be more often associated with both PVF and CBVL than other HL causes. The odds of having delays in 4 developmental milestones (head holding, sitting, standing with support, and independent walking) were higher in both PVF and CBVL (eg, head-holding odds ratios: 2.55 and 4.79) compared with NVF, and the age of achieving these milestones was higher in CBVL than PVF (eg, head holding: 7.33 vs 4.03 years; *P* < .001). All 4 developmental milestones were associated with the degree of vestibular impairment.

**Conclusions and Relevance:**

This cohort study found that among children with profound HL, vestibular impairment was prevalent, varied according to HL origin, and associated with posturomotor development; while all developmental milestones were associated with vestibular impairment severity, not all HL causes were associated with vestibular impairment severity. Children with profound HL may benefit from complete vestibular assessment before cochlear implantation, which would support early and adapted management, such as physical therapy for CBVL and cochlear implantation strategy.

## Key Points

**Question:**

How common is vestibular impairment in children with profound hearing loss, and is vestibular impairment associated with developmental delays?

**Findings:**

In this cohort study of 592 children with bilateral profound hearing loss, vestibular impairment was found in 44% of the children. A delay in achieving developmental milestones was associated with vestibular impairment severity.

**Meaning:**

Findings of this study suggest that assessing vestibular function in all children prior to cochlear implantation is necessary in prescribing timely adapted vestibular rehabilitation and proposing cochlear implantation strategies for preserving vestibular function.

## Teaser

This cohort study assesses the association between vestibular function loss and ages at which head holding, sitting, standing with support, and independent walking were reached among children who were candidates for a cochlear implant.

## Introduction

Although the diagnosis and management of hearing loss (HL) in children have undergone substantial changes in the past 30 years, vestibular function assessment in this population has been considered only in the past 15 years.^1,2,3,4,5,6,7,8,9,10,11^ A large number of tests are available for the identification of HL origin, the choice and combination of which can be guided by the patient’s medical history and known clinical syndromes. For example, bilateral HL with syncopal episodes and prolonged QT interval on the electrocardiogram is suggestive of Jervell and Lange-Nielsen syndrome, while depigmentation of the iris, skin, or hair and eye heterochromia are suggestive of Waardenburg syndrome. For congenital cytomegalovirus (CMV), as soon as infection is suspected (from positive maternal serology result during pregnancy, syndromic neonate at birth, and early progressive HL), polymerase chain reaction should be performed on saliva, urine, or blood in the first 2 weeks of life ideally or on Guthrie dried blood spots later. The risk of neurosensory sequelae will be established if the infection occurred before the 20th week of gestation, after analysis of the maternal serum samples harvested during the pregnancy.^12,13,14^ A complete bilateral vestibular loss (CBVL) is suggestive of possible Usher syndrome, which should lead to a search for retinitis pigmentosa by an ophthalmologist, followed by an electroretinogram and a genetic diagnosis. A syndrome involving coloboma, choanal atresia, and absence of semicircular canals on the computed tomography scan is suggestive of CHARGE syndrome, which can be confirmed by the genetic identification of a *CDH7* sequence variant. Vestibular assessment, however, is rarely included in HL etiologic screening.

In the literature, the incidences of different HL causes are highly variable according to study cohort size and population characteristics.^15,16,17,18,19,20,21,22^ The most common causes for bilateral HL in children are CMV infection and genetic causes. Genetic causes represent 50% of pediatric HL, with approximately 70% being nonsyndromic and 30% being syndromic genetic cases. Since genetic HL is more often bilateral than unilateral (2.5 times more often), genetic testing plays a greater role in the evaluation of bilateral HL.^20^ The widespread availability of genetic testing has changed the diagnostic landscape of HL, particularly in nonsyndromic cases. With modern testing methods, a genetic cause can be identified in approximately 50% of patients with bilateral HL.^20^ It has been reported^23^ that in 100 recipients of pediatric cochlear implant who underwent genetic testing, 48% were identified as having causative genetic sequence variations, affecting 17 genes. The most common of these genes were *GJB2,* connexin 26 (36%), *SLC26A4* (13%), *MYO15A* (8%), and *MYO7A* (8%).

Few and relatively recent studies have focused on vestibular impairment in pediatric HL,^1,2,3,4,5,6,7,8,9,10^ and, to our knowledge, no association has been reported between HL origin and vestibular impairment, particularly nonsyndromic HL. Assessing vestibular function is of particular interest since it has been found that vestibular impairment has an adverse implications for the postural, motor, and cognitive development in children.^3,23,24,25^ Moreover, among children with a cochlear implant, those with vestibular impairment have worse outcomes due to fall-related implant dysfunctions^2^ and learning disabilities directly associated with their vestibular impairment.^24^

Yet, the decision for cochlear implantation is rarely based on vestibular assessment as a complement to audiological testing.^1,5^ Evaluating vestibular function prior to cochlear implantation could also help in deciding which strategy to apply between simultaneous bilateral and sequential bilateral cochlear implant.^1,5^ The aim of this study was to identify the prevalence of vestibular impairment according to HL origin and to assess the association between vestibular impairment and delayed posturomotor development in a large cohort of children with profound HL.

## Methods

### Patients, Setting, and Study Design

This retrospective single-center cohort study was carried out using data collected in Paris, France, between January 1, 2009, and December 31, 2019. The Comité de Protection des Personnes Ouest III deemed the study exempt from review and waived the informed consent requirement because of the retrospective nature of the study. We followed the Strengthening the Reporting of Observational Studies in Epidemiology (STROBE) reporting guideline.

All pediatric patients included in the study ranged in age from 2 months to 8 years, had bilateral profound HL (loss >90 dB HL), and were candidates for a cochlear implant. Consequently, they all underwent systematic audiological and vestibular testing at a pediatric referral center prior to any cochlear implant decision.^1^ The vestibular clinical examination consisted of a complete neuro-otological and vestibular battery of tests, including standing on the floor and on a postural pad with eyes open and eyes closed, oculomotor examination (gaze stability, pursuit, saccades, vergence, optokinetic responses, and oculomotor field), inhibition of the vestibulo-ocular reflex (VOR) by eye fixation on a target during chair rotation, head impulse test, and videoscopy (for eye counter-rolling reflex, spontaneous nystagmus, induced nystagmus with positioning, head-shaking test, and VOR during chair rotation). This examination was complemented by a battery of vestibular tests covering the full range of sensitivity of the semicircular canals (from low to high head-rotation velocities) and otolith organs. For semicircular canal function assessment, bithermal caloric test, video head impulse test with remote camera, and earth vertical axis rotation test were used. For otolith function, cervical vestibular evoked myogenic potential test with bone conduction was used. All these tests were adapted for use in children and are routinely performed at the pediatric referral center from the age of 3 months; normative data in children and pediatric protocols are available for video head impulse test, earth vertical axis rotation test, and cervical vestibular evoked myogenic potential test.^26,27,28,29,30,31^ For the bithermal caloric test, the pediatric protocol differs from the adult protocol by irrigation in the seated position; videoscopy with glasses not attached to the head; and replacement of the shutter by a 20-diopter lens to suppress VOR inhibition by fixation, avoid complete darkness, and stimulate attention by the brief and random appearance of a small toy through the lens. The video image is displayed on a large screen, and the frequency of the nystagmus quick phases is counted between 60 and 90 seconds after irrigation onset. This nystagmus frequency was used to calculate the relative reflectivity and directional preponderance (normal values <15%).^32,33^

The vestibular clinical examination and vestibular testing were performed by ear, nose, and throat specialists assisted by a technician. All clinicians were highly experienced in vestibular testing in children.

All children were screened for HL causes, including ophthalmological and cardiovascular examinations, urine dipsticks, and radiological imaging (magnetic resonance imaging and computed tomography scan). The genetic screening was not systematically performed since some families declined it.

### Data Collection

Data on HL origin, vestibular evaluation, and developmental milestones were collected from the patients’ medical records. Three groups were then defined according to their vestibular function status: normal vestibular function (NVF), partially impaired vestibular function (PVF), and CBVL.

Four developmental milestones of posturomotor control (head holding, sitting, standing with support, and independent walking) and the age at which they were reached were chosen to measure the association between vestibular impairment and development.^12,34^ These data were collected from the children’s medical records, which are mandatory in France and follow the children from birth to adulthood.

### Statistical Analysis

The mean differences between the 3 groups of children were evaluated by independent 1-way analysis of variance for continuous variables and χ^2^ tests for categorical variables. Generalized logistic models were used to identify the factors associated with the 2 groups with vestibular impairment (PVF and CBVL). The models’ ability to discriminate between NVF and PVF or between NVF and CBVL was assessed through the receiver operating characteristic (ROC) curve and area under the ROC curve (AUC) analyses.

Regarding the age-related variables, a 2-month increment was selected for calculating the odds ratios (ORs). For example, the OR for head-holding age obtained when comparing the PVF to the NVF group (OR, 2.55) signified that for every 2-month increase in age at achieving head holding, the odds of having PVF were 2.55 times higher than the odds of having NVF. This interpretation applied to all of the other ORs of the age-related variables, with the severity of the vestibular impairment.

The ROC curve analysis focused on the performances of the logistic models on several parameters characterizing the vestibular impairments. Sensitivity and specificity measures quantified the proportions of true-positive cases (ie, instances when the model correctly identifies individuals with vestibular impairments) and true-negative cases (ie, instances when the model correctly identifies individuals without vestibular impairments), respectively, correctly identified by the models. Perfect model performance would yield a ROC curve showing 100% sensitivity and 100% specificity, with an AUC of 1. Conversely, a test with no discriminatory power, essentially random guessing, would result in an AUC of 0.5. The AUC represents the probability that the model will correctly classify a randomly chosen positive case higher than a randomly chosen negative case. Higher AUC values indicate better discrimination, with values closer to 1 indicating superior performance.

Two-sided *P* < .05 indicated statistical significance. Data analyses were conducted between January and June 2023 using RStudio 4.2.1 (RStudio).

## Results

A total of 592 children were included in the study. Of these patients, 284 (48.0%) were females and 308 (52.0%) were males, with a mean (SD) age of 38 (34) months. These patients were classified as follows: the NVF group included those with normal responses to all of the vestibular tests, the PVF group included those with no detectable response or abnormal responses to at least 1 test, and the CBVL group comprised those with no response to any of the tests (Table 1). Due to the classification used, the PVF group included children with a wide range of partial vestibular deficits, from very mild to severe.

**Table 1.** Classification Criteria for Vestibular Functions

Vestibular function evaluation	Criteria of selection	HIT and vHIT	EVAR test	Caloric bithermal test	cVEMP test
NVF	Normal responses to all the tests	No catch-up saccades present and gain ≥0.8	Maximum slow-phase velocity and time constant (function of age)	Quick-phase frequency (l Hz) and relative hypovalence and directional preponderance *≤*15%	Amplitude ratio (P-N/EMG) (function of age)
CBVL	No detectable response to any of the tests	Presence of catch-up saccades and gain *≤*0.2	No response	No response at 20 °C irrigation in both ears	No detectable response at maximum stimulation level (110 dB HL = 98 dB nHL)
PVF	No detectable response or abnormal response to at least 1 test	No detectable response or abnormal response to at least 1 test	No detectable response or abnormal response to at least 1 test	No detectable response or abnormal response to at least 1 test	No detectable response or abnormal response to at least 1 test

Abbreviations: CBVL, complete bilateral vestibular loss; cVEMP, cervical vestibular evoked myogenic potential; dB, decibel; dB nHL, decibels normalized hearing level; EMG, electromyogram; EVAR, earth vertical axis rotation; HIT, head impulse test; HL, hearing loss; NVF, normal vestibular function; PVF, partially impaired vestibular function; vHIT, video head impulse test.

Among 266 cases with a confirmed HL origin (266), 45.1% (120) had HL of genetic origin, of which 50.0% (60) were syndromic (the most frequent being Usher and Waardenburg syndromes) and 50.0% (60) were nonsyndromic (the most frequent being associated with connexin 26). Infectious origin accounted for 12.5% (74 of 592) of HL cases, the majority of which (70.3% [52]) had CMV. Inner ear malformations accounted for 11.3% (67 of 592) of HL cases, 83.6% (56) of which had nonsyndromic or 16.4% (11) syndromic origin. Perinatal distress (2.0% [12 of 592]) and toxic (0.7% [4 of 592]) causes of HL were the least prevalent. The HL origin was not documented in 326 cases (55.1%) (Table 2).

**Table 2.** Hearing Loss (HL) Origin and Vestibular Function Status of Candidates for Cochlear Implantation (n = 592)

HL origin	Sample size	No. (%)		
		NVF	PVF	CBVL
Genetic nonsyndromic	60	45 (75.0)	15 (25.0)	0
Connexin 26	41	31 (75.6)	10 (24.4)	0
Nonsyndromic, no connexin 26	19	14 (73.7)	5 (26.3)	0
Otoferlin	1	1 (100)	NA	NA
Mitochondrial disease	1	NA	1 (100)	NA
Consanguinity and familial HL (17)	17	13 (76.5)	4 (23.5)	0
Genetic syndromic	60	13 (21.6)	34 (56.6)	13 (21.6)
Usher^a^	22	1 (4.5)	12 (54.5)	9 (40.9)
Waardenburg^a^	20	8 (40.0)	10 (50.0)	2 (10.0)
Rare syndromic	18	5 (27.8)	11 (61.1)	2 (11.1)
Jervell and Lange-Nielsen (SQTL)	2	1 (50.0)	NA	1 (50.0)
Alport (glomerulonephritis)^a^	1	NA	1 (100)	NA
Bartter type IV (tubulopathy)	1	1 (100)	NA	NA
BOR^a^	2	1 (50.0)	1 (50.0)	NA
Down (trisomy 21)	2	1 (50.0)	NA	1 (50.0)
CHARGE	1	NA	1 (100)	NA
Di Georges (deletion 22q11)	1	NA	1 (100)	NA
Duplication 22 q 11.2	1	NA	1 (100)	NA
DOOR	1	NA	1 (100)	NA
Duane with Mondini	1	NA	1 (100)	NA
Hurler	1	NA	1 (100)	NA
Klinefelter (XXY)	1	NA	1 (100)	NA
Polymalformation	2	1 (50.0)	1 (50.0)	NA
Brown-Vialetto-Van Laere	1	NA	1 (100)	NA
Inner ear malformation	67	28 (41.7)	36 (53.7)	3 (4.5)
Nonsyndromic	56	28 (50.0)	28 (50.0)	0
Syndromic^a^	11	0	8 (72.7)	3 (27.2)
Infectious	74	23 (31.0)	43 (58.1)	8 (10.8)
CMV	52	16 (30.7)	30 (57.6)	6 (11.5)
Meningitis	17	7 (41.1)	9 (52.9)	1 (5.8)
Various infections	5	0	4 (80.0)	1 (20.0)
Mumps	1	0	1 (100)	0
Materno-fetal infection	2	0	1 (50.0)	1 (50.0)
Kabuki	1	0	1 (100)	0
Sarcoidosis	1	0	1 (100)	0
Toxic	4	1 (25.0)	3 (75.0)	0
Chemotherapy	2	0	2 (100)	0
Aminosid	1	1 (100)	0	0
Neonatal jaundice	1	0	1 (100)	0
Perinatal distress	12	3 (25.0)	7 (58.3)	2 (16.7)
Undetermined	326	216 (66.3)	99 (30.3)	11 (3.4)

Abbreviations: BOR, Branchiootorenal; CBVL, complete bilateral vestibular loss; CHARGE, involving coloboma, heart defects, atresia choanae, retardation of growth and development, genitourinary disorders, and ear anomalies; CMV, cytomegalovirus; DOOR, deafness, onychodystrophy, osteodystrophy, and mental retardation; NA, not applicable; NVF, normal vestibular function; PVF, partially impaired vestibular function; SQTL, splicing quantitative trait locus.

^a^ Note that within the 67 malformations of the inner ear identified (Mondini, DAV, Gusher), 56 were isolated without any clinical associated syndrome and 11 were part of an identified syndrome: 8 Waardenburg, 1 Alport, 1 BOR, and 1 Usher type 1 syndromes.

### Prevalence of Vestibular Impairment by HL Origin

Vestibular impairment was found in 263 children (44.4%), of whom 34 (5.7%) had CBVL. Vestibular impairment was symmetrical in 526 children (88.9%).

The HL causes associated with the highest proportion of CBVL were genetic syndromic and infectious (6 of 8 infections due to CMV). Most patients (78.3% [47]) with genetic syndromic HL showed vestibular impairment, of whom 56.7% (34) presented with PVF and 21.7% (13) with CBVL. Among children with Usher syndrome (n = 22), 95.5% (21) had a vestibular impairment, with 40.9% (9) having CBVL. Among children with Waardenburg syndrome (n = 20), 60.0% (12) had a vestibular impairment, with 10.0% (2) having CBVL. Among patients with genetic nonsyndromic HL (n = 60), 0 had CBVL and 25.0% (15) had PVF (Table 2).

Thirty-six children (69.2%) with CMV infections showed vestibular impairment, including 57.7% (30) with PVF and 11.5% (6) with CBVL. Among patients with HL due to nonsyndromic inner ear malformation (n = 56), 50.0% (28) had NVF and 0 had CBVL. Conversely, among children with syndromic inner ear malformation (n = 11), 27.3% (3) presented with CBVL. Among children with undetermined HL origin (n = 326), 30.4% (99) had PVF and 3.4% (11) CBVL (Table 2). Results of the χ^2^ test suggest an association between vestibular impairment and HL origin (χ^2^ = 102.27, df = 12; *P* < .001).

### Factors Associated With Vestibular Impairment

When comparing the NVF, PVF, and CBVL groups on the basis of age of acquisition of the 4 developmental milestones, the 1-way analysis of variance revealed significant differences between the 3 groups for each continuous variable tested. The age of acquisition for the 4 posturomotor milestones was significantly higher for CBVL vs NVF and PVL vs NVF (eg, head holding: 7.33 vs 3.27 years and 4.03 vs 3.27 years; *F* statistic = 40.1; *P* < .001) and for CBVL vs PVL (eg, head holding: 7.33 vs 4.03 years; *F* statistic = 40.1; *P* < .001) (Figure).

**Figure.**
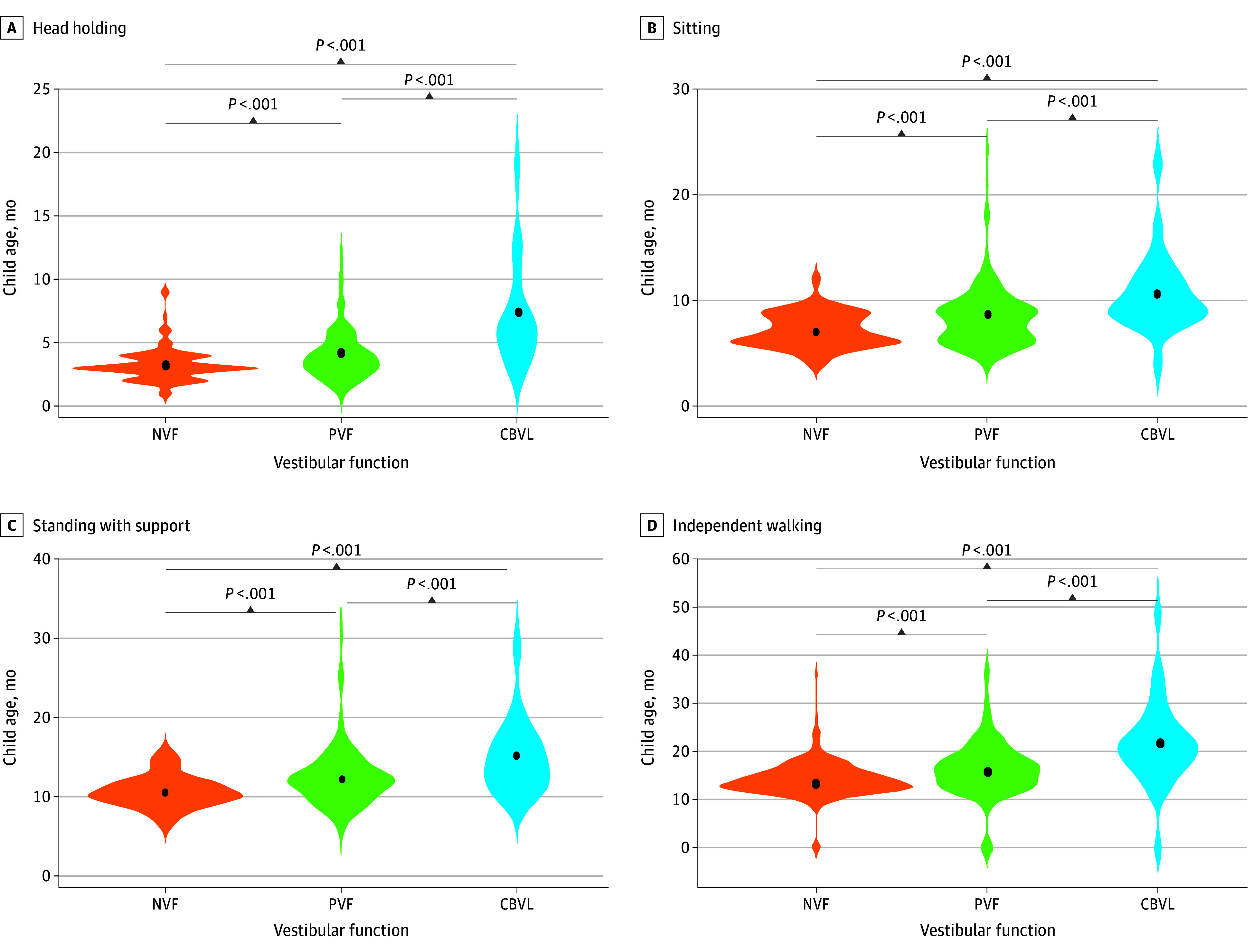
Association Between Age of Developmental Milestones Achievement and Vestibular Status CBVL indicates complete bilateral vestibular loss; NVF, normal vestibular function; PVF, partially impaired vestibular function. Significant mean differences are shown (*P* < .001). Black dots inside the violins represent the mean age of developmental milestone achievement for each vestibular status.

Generalized logistic models showed that in terms of HL origin, when compared with NVF, genetic syndromic (OR, 1.21; 95% CI, 1.03-1.48), genetic nonsyndromic (OR, 1.19; 95% CI, 1.13-2.77), and inner ear malformation (OR, 1.79; 95% CI, 0.99-2.64) were more often associated with PVF than other HL causes. Genetic syndromic (OR, 1.29; 95% CI, 1.08-1.31) and infectious origin (OR, 3.28; 95% CI, 3.48-4.11) emerged as more often associated with CBVL (Table 3). According to the logistic models, the probability of having CBVL rather than NVF was higher than the probability of having PVF rather than NVF, and the delay in acquisition of all 4 posturomotor milestones was more pronounced in CBVL.

**Table 3.** Results of the Generalized Logit Model for the Association of Vestibular Impairment With Hearing Loss (HL) Origin^a^

Vestibular impairment and HL origin	OR (95% CI)
**PVF**	
Genetic syndromic	1.21 (1.03-1.48)^b^
Genetic nonsyndromic	1.19 (1.13-2.77)^b^
Infectious	0.12 (0.68-0.89)
Inner ear malformation	1.79 (0.99-2.64)^b^
Perinatal distress	0.30 (0.28-1.31)
Toxic	0.06 (0.01-0.33)
**CBVL**	
Genetic syndromic	1.29 (1.08-1.31)^b^
Genetic nonsyndromic	7.59 (7.34-7.78)
Infectious	3.28 (3.48-4.11)^b^
Inner ear malformation	7.48 (7.02-8.20)
Perinatal distress	2.10 (1.05-2.54)
Toxic	4.66 (4.10-5.89)

Abbreviations: CBVL, complete bilateral vestibular loss; NVF, normal vestibular function; OR, odds ratio; PVF, partially impaired vestibular function.

^a^ The reference categorical variable for running the generalized logit model was undetermined HL origin. The model compared PVF to NVF and CBVL to NVF (with NVF as the reference).

^b^ Significant *P* value.

Regarding the age-related variables, the OR calculated with a 2-month increment when comparing PVF to NVF groups for head-holding age was 2.55 (95% CI, 0.40-0.71). This OR signified that for every 2-month increase in the age of achieving head holding, the odds of having PVF were 2.55 times higher than the odds of having NVF. This calculation applied to all of the other ORs of the age-related variables. Based on this analysis, the odds of having delays in all 4 posturomotor developmental milestones were higher in both PVF (head holding OR, 2.55 [95% CI, 0.40-0.71]; sitting OR, 2.24 [95% CI, 0.13-0.35]; standing with support OR, 2.04 [95% CI, 0.02-0.12]; independent walking OR, 2.23 [95% CI, 0.18-0.29]) and CBVL (head holding OR, 4.79 [95% CI, 3.67-4.83]; sitting OR, 5.77 [95% CI, 5.46-5.15]; standing with support OR, 4.27 [95% CI, 4.16-4.38]; independent walking OR, 5.45 [95% CI, 5.33-5.57]) groups and consistently increased with the severity of the vestibular impairment (Table 4).

**Table 4.** Results of the 4 Generalized Logit Models for the Association of Vestibular Impairment With 4 Developmental Milestones^a^

Developmental milestone and vestibular impairment	OR (95% CI)
**Head-holding age**	
NVF	1 [Reference]
PVF	2.55 (0.40-0.71)
CBVL	4.79 (3.67-4.83)
**Sitting age**	
NVF	1 [Reference]
PVF	2.24 (0.13-0.35)
CBVL	5.77 (5.46-5.15)
**Standing-with-support age**	
NVF	1 [Reference]
PVF	2.04 (0.02-0.12)
CBVL	4.27 (4.16-4.38)
**Independent-walking age**	
NVF	1 [Reference]
PVF	2.23 (0.18-0.29)
CBVL	5.45 (5.33-5.57)

Abbreviations: CBVL, complete bilateral vestibular loss; NVF, normal vestibular function; OR, odds ratio; PVF, partially impaired vestibular function.

^a^ The models compared PVF to NVF and CBVL to NVF for each age-related variable (with NVF as the reference). All ORs have significant *P* values.

The ROC curves identified the age-related variables that differentiated children with vestibular impairment from children with NVF. Regarding the head-holding age, the AUC was 0.688, and a cutoff of 4.5 months showed a sensitivity of 78% and a specificity of 87%. Sitting age had an AUC of 0.745, and a cutoff of 7 months had a sensitivity of 73% and a specificity of 89%. Age for standing with support had an AUC of 0.833, and a cutoff of 9.5 months showed a sensitivity of 79% and a specificity of 91%. Independent-walking age had an AUC of 0.871, and a cutoff of 11.25 months demonstrated a sensitivity of 71% and a specificity of 84%.

## Discussion

In this large cohort of children with profound HL who were candidates for cochlear implants, vestibular impairment was found in 44.4% and was complete in 5.7% of these children. Certain HL origins (namely, genetic syndromic and infectious) appeared to be more likely factors in CBVL than other causes. Posturomotor development also appeared to be associated with vestibular impairment, as the age of acquisition of 4 developmental milestones increased with the degree of vestibular impairment. Overall, although all developmental milestones were factors in vestibular impairment, not all HL causes were associated with the vestibular impairment severity.

The proportion of vestibular impairment found in the present study, which was symmetric in most cases, is slightly lower than the 51% reported in a previous study.^1^ Moreover, the proportion of children with CBVL was 3 times lower than in the previous study (20%). However, the higher percentage could represent an overestimation of CBVL cases since the vestibular testing methods at the time of the study were less sensitive.^1^ Regarding the distribution of HL origin, it is difficult to compare the present results with those in the literature, partly due to the advancement in technologies, which now allow for better screening of CMV and genetic causes, but also because of differences in the size and characteristics of the populations studied, as most of the studies did not focus on children or on cochlear implant candidates with profound HL.^15,16,17,18,19,20,21,22,34,35,36^ In the present cohort, the main causes of profound HL in children were genetic and infectious. Of note, the proportion of children with unidentified HL origin was high (55.1%), but it was close to the range of 33% to 48% reported in the meta-analysis by Petersen et al.^35^ This finding could be explained by the fact that, in France, genetic testing for HL in children cannot systematically be performed because the decision is left to the parents. This condition may have induced an underestimation of the proportion of genetic HL origin in the present cohort.

Although some HL causes are frequently associated with severe vestibular impairment (eg, CMV), some children with such infectious origin will be spared of vestibular loss and others will not. Several physiopathological mechanisms may explain these differences in vestibular vulnerability. For instance, CMV infection is associated with severe but variable neurosensory sequelae (92% of vestibular impairment with 33% of CBVL^1^), but only for a small proportion of children with infection (12%-14%).^1^ Different factors may explain the variable severity of the HL and vestibular loss in children with CMV, such as the initial viral load that penetrates unevenly the inner ear structures, the period of pregnancy at which the fetus was infected, and the immune system strength of both the mother and child. In genetic HL, the consequences of a sequence variant depend on gene type and location, as demonstrated by the genotype-phenotype correlations observed in Usher syndrome.^37^ Connexin 26–related HL is rarely associated with vestibular impairment (24.4% [10] of patients with PVF and 0 with CBVL in the present study [Table 2]), which can be explained by the sequence variation affecting the synthesis of a protein that is not present in the vestibule but in the cochlea.^38^

The association of vestibular impairment with the age of achieving independent walking in children has previously been reported in the context of vestibular impairment severity.^1,7,23,25,27,39,40,41^ In the present study, the ages of all 4 developmental milestones were substantially delayed in both CBVL and PVF groups compared with the NVF group and were even more delayed for CBVL. Moreover, the analysis demonstrated that the delayed age of developmental milestones is more often associated with CBVL than PVF. These results are consistent with those of Janky et al,^7^ who found that, in a cohort of children with different degrees of HL, parental concern for gross motor developmental delay was the parameter that best differentiated children with vestibular loss from children with normal vestibular function.^7^ Together, these findings suggest that delayed posturomotor development in children with HL is often a direct consequence of vestibular impairment, particularly CBVL. Nonetheless, nonvestibular comorbidities such as musculoskeletal dysmorphia or other organ involvement and neurological impairments can also contribute to delayed motor development. Another diagnostic complication is that the delays in posturomotor development associated with CBVL resemble symptoms of some neurological disorders (axial hypotonia, no parachute reflex, and no fall anticipation). We therefore recommend that children with profound HL who show a delay in posturomotor development undergo a vestibular evaluation before being diagnosed with a neurological disorder. Furthermore, given the evidence that early vestibular rehabilitation is associated with improved psychomotor development in children with vestibular impairment,^41,42,43^ early detection of vestibular impairment is highly recommended.

It is essential to ascertain precisely, using a comprehensive cognitive, neurological, and sensorimotor assessment, the deficits and expectations for each child. This comprehensive assessment would enable clinicians to target cognitive, neurological, or sensorimotor difficulties and individualize the therapeutic rehabilitation program, the success of which depends on providing counseling to and empowering parents and caregivers.^41,42,43^

### Limitations

This study has several limitations. First, the inability to link some HL causes with vestibular impairment could be due to a lack of statistical power given the small number of patients presenting with perinatal distress and toxic causes, for example. Second, the study included only children with profound HL who were candidates for cochlear implant but not children with other levels of HL who were not candidates for cochlear implant, and the prevalence of vestibular impairment may be different compared with the prevalence in the present study’s cohort. This exclusion was even more relevant since it has been shown that the severity of vestibular impairment increases with the degree of HL.^7^ Third, the PVF group had vestibular impairments of varying severity (from very mild to very severe). Defining subgroups of vestibular impairment within the PVF group would enable the refinement of the association between vestibular impairment and posturomotor development.

## Conclusions

The findings of this cohort study showed that vestibular impairment was prevalent in pediatric patients with bilateral profound HL who were candidates for cochlear implant. Furthermore, both partial and complete bilateral vestibular impairments were associated with delays in posturomotor development. Although some HL causes had different vestibular impairment severity, a delay in all 4 developmental milestones could be viewed as an argument for vestibular impairment, particularly CBVL. This finding suggests that children with profound HL benefit from a complete vestibular assessment before cochlear implantation, which would put in place early and adapted management, such as vestibular substitution therapy for CBVL and cochlear implant strategy.

## Notes

Supplement.
Data Sharing Statement
