# Supplementary material for: Vestibular Impairment and Postural Development in Children With Bilateral Profound Hearing Loss
Source: JAMA Netw Open. 2024 May 23;7(5):e2412846. doi: 10.1001/jamanetworkopen.2024.12846 (PMC11117085; doi:10.1001/jamanetworkopen.2024.12846)
Supplement: Supplement. — Data Sharing Statement [file jamanetwopen-e2412846-s001.pdf]

Data Sharing Statement

Wiener-Vacher. Vestibular Impairment and Postural Development in Children With Bilateral Profound Hearing Loss. *JAMA Netw Open*. Published May 23, 2024.
doi:10.1001/jamanetworkopen.2024.12846

Data
Data available: Yes
Data types: Deidentified participant data
How to access data: Sylvette.wiener@gmail.com
When available: With publication

Supporting Documents
Document types: Statistical/analytic code
How to access documents: https://github.com/mcampi111/VestibularLoss/tree/main
When available: With publication

Additional Information
Who can access the data: Researchers whose proposed use of the data has been approved
Types of analyses: medical research
Mechanisms of data availability: after approval of a proposal
